# Supplementary material for: An Analysis of the Effectiveness and Safety of Upadacitinib in the Treatment of Inflammatory Bowel Disease: A Multicenter Real-World Study
Source: Biomedicines. 2025 Jan 14;13(1):190. doi: 10.3390/biomedicines13010190 (PMC11761900; doi:10.3390/biomedicines13010190)
Supplement: Supplementary file 1 [file biomedicines-13-00190-s001.zip › Supplemental Table.pdf]

Supplemental Table 1. Comparison of baseline characteristics and clinical outcomes between patients with and without endoscopic evaluation

Variables	CD *P*-value	UC *P*-value
**BMI**	0.704	0.524
**Age**	0.201	0.688
**Disease duration**	0.427	0.610
**Disease location**	0.737	0.564
**Disease behavior**	0.737	-
**Disease activity**	1.000	0.949
