# Supplementary material for: An Analysis of the Effectiveness and Safety of Upadacitinib in the Treatment of Inflammatory Bowel Disease: A Multicenter Real-World Study
Source: Biomedicines. 2025 Jan 14;13(1):190. doi: 10.3390/biomedicines13010190 (PMC11761900; doi:10.3390/biomedicines13010190)

In the UC cohort, one case of asymptomatic pulmonary embolism was reported in a 60-year-old female patient treated with upadacitinib. The patient had a 4-year history of UC, a BMI of 18.99 kg/m², and no history of chronic diseases such as hypertension or coronary heart disease. She was a non-smoker. Previous treatments with infliximab and vedolizumab were ineffective. In July 2023, the patient started oral upadacitinib at an initial dose of 45 mg, followed by a maintenance dose of 15 mg for 8 weeks, achieving mucosal healing. In January 2023, the patient experienced disease relapse, and a CT scan revealed multiple pulmonary embolisms without symptoms such as chest pain, hemoptysis, or dyspnea. The etiology of the thrombosis was considered to be related to the hypercoagulable state during the active phase of UC and the potential association with upadacitinib. An interventional medicine consultation was requested, and anticoagulant therapy was recommended. However, the patient developed acute severe UC with massive bleeding, necessitating laparoscopic total colectomy with ileal pouch-anal anastomosis (single pouch) and percutaneous abdominal drainage. The patient was followed up by the interventional medicine department post-operatively. In January 2024, the patient's Mayo score was as follows: stool frequency: 3; rectal bleeding: 2; endoscopic subscore: 3; physician's global assessment: 3; total Mayo score: 11. Baseline Mayo scores and D-dimer levels were unavailable as the patient had previously received treatment at another hospital. The patient's imaging data are shown in the **Supplementary Figure 3**.

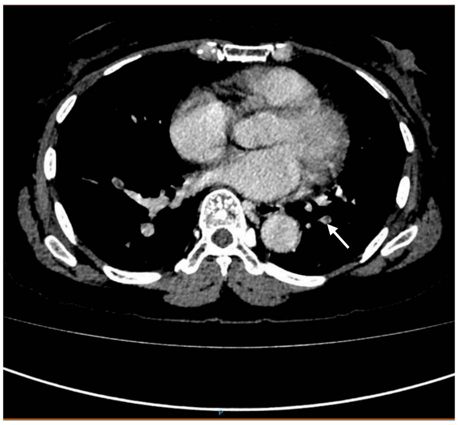
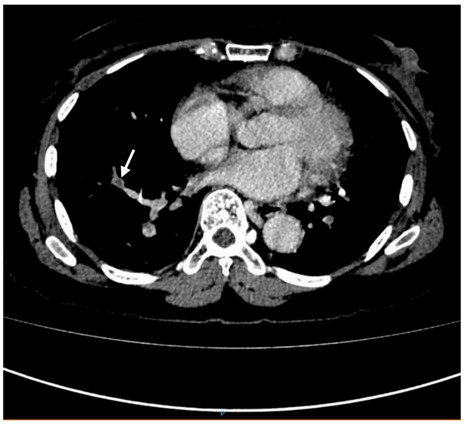
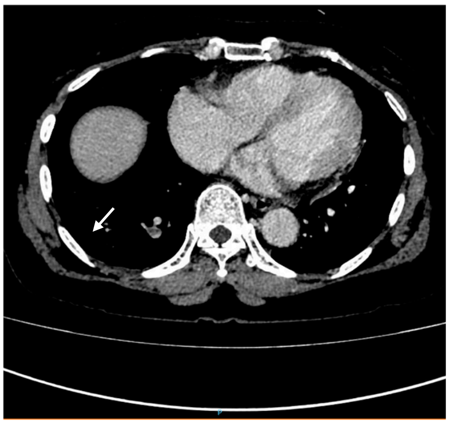

Supplement: Supplementary file 1 [file biomedicines-13-00190-s001.zip › Supplemental Text.pdf]
