# Supplementary figures and images for: An Analysis of the Effectiveness and Safety of Upadacitinib in the Treatment of Inflammatory Bowel Disease: A Multicenter Real-World Study
Source: Biomedicines. 2025 Jan 14;13(1):190. doi: 10.3390/biomedicines13010190 (PMC11761900; doi:10.3390/biomedicines13010190)

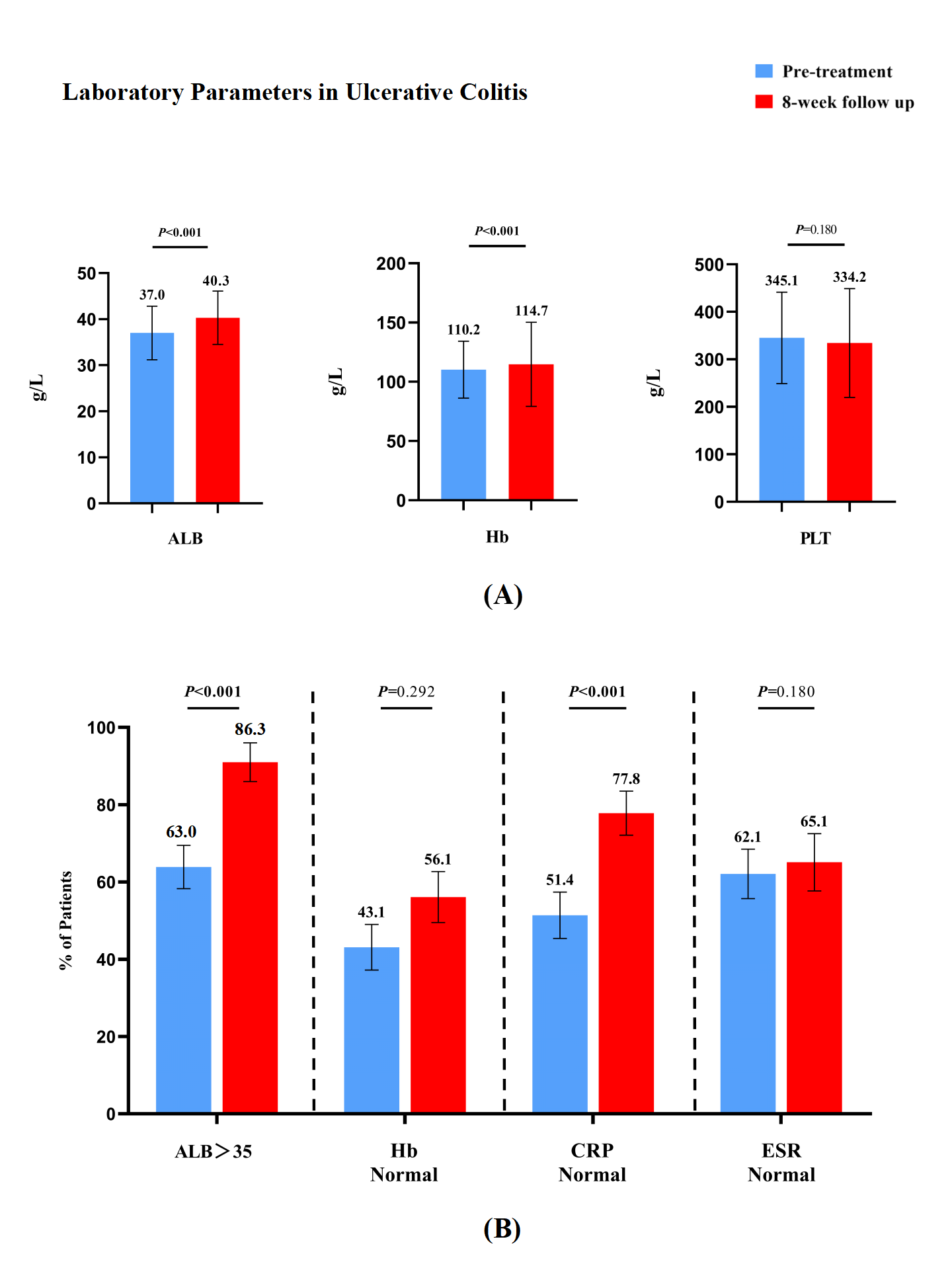

Supplement: Supplementary file 1 [file biomedicines-13-00190-s001.zip › Supplemental Figure 1.tif]

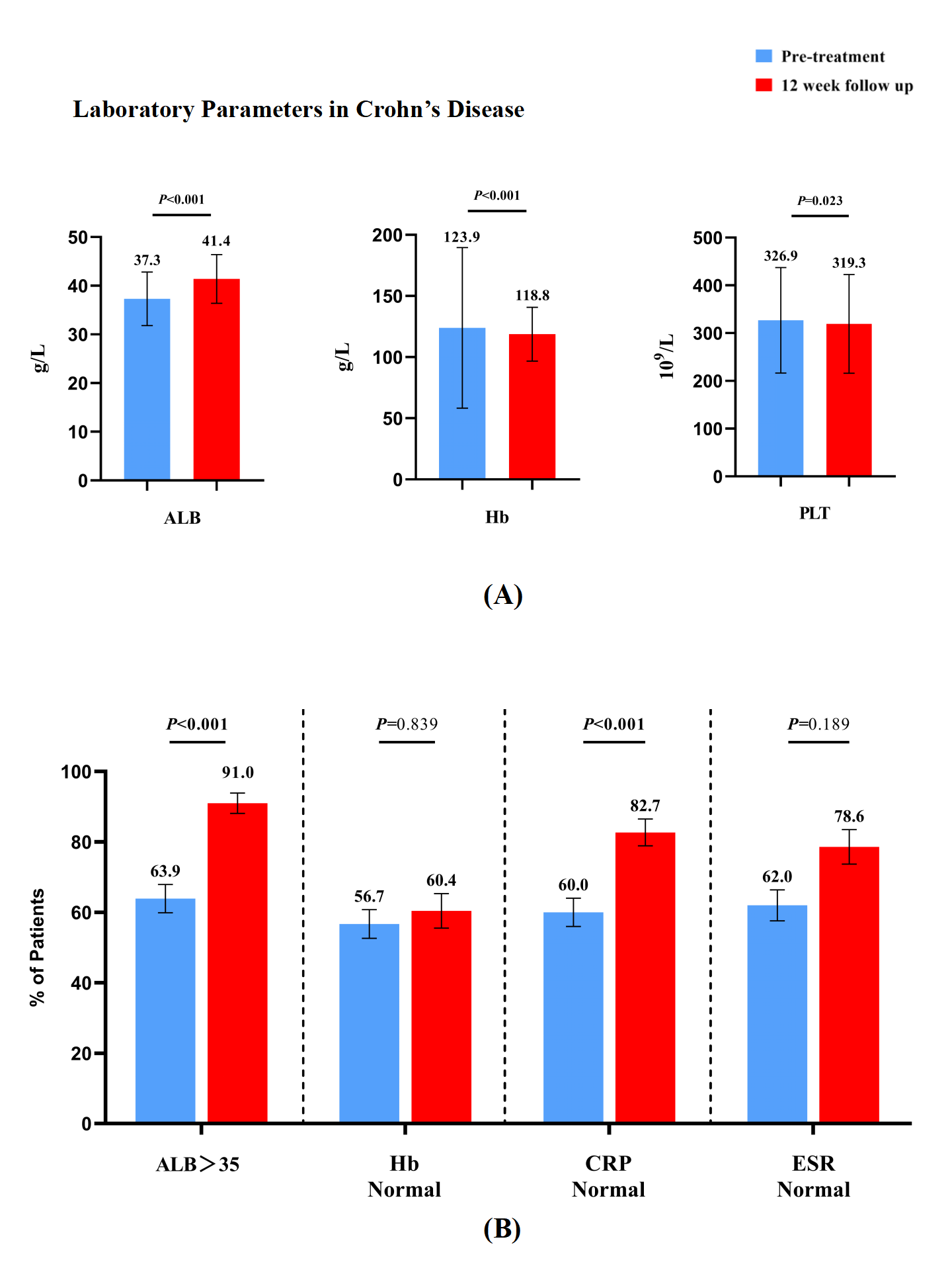

Supplement: Supplementary file 1 [file biomedicines-13-00190-s001.zip › Supplemental Figure 2.tif]

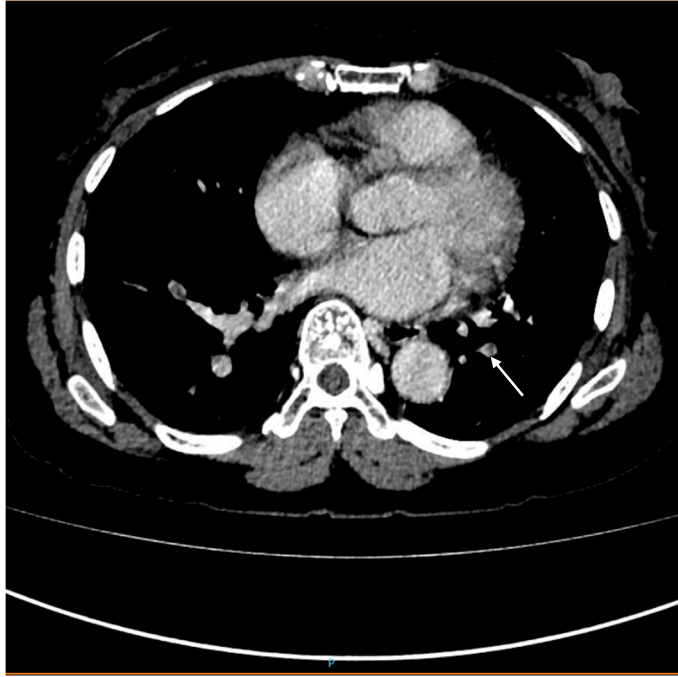

(A)

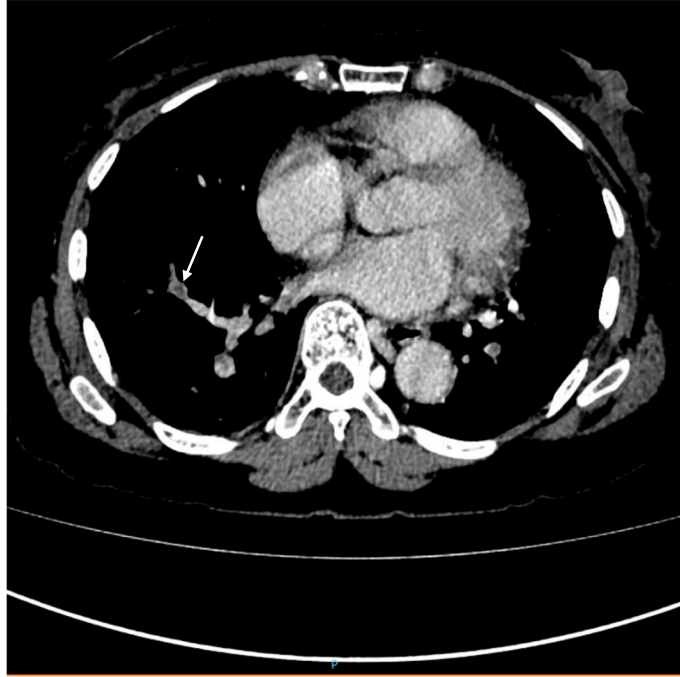

(B)

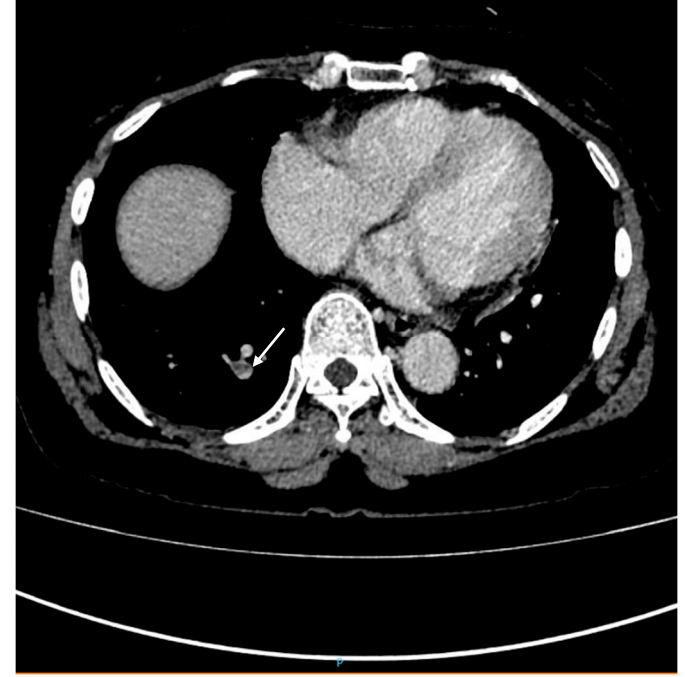

(C)

Supplement: Supplementary file 1 [file biomedicines-13-00190-s001.zip › Supplemental Figure 3.pdf]

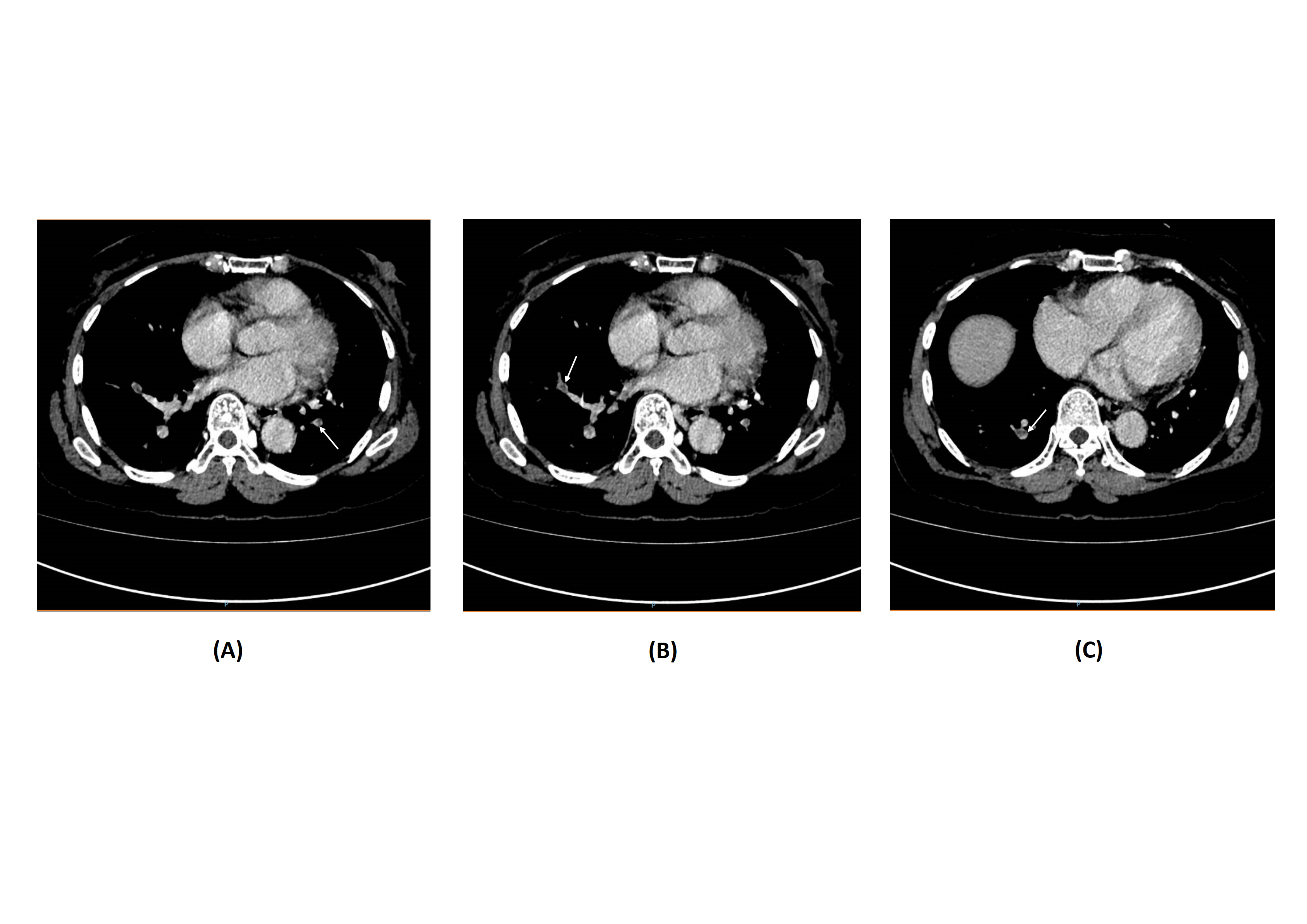

Supplement: Supplementary file 1 [file biomedicines-13-00190-s001.zip › Supplemental Figure 3.tif]
